# Anatomical and Pathological Observations

**Published:** 1845-07

**Authors:** 


### Anatomical and Pathological Observations. By *John Goodsir,* F.R.S.E., Demonstrator of Anatomy in the University of Edinburgh, and *Harry D. S. Goodsir,* M.W.S., Conservator of the Museum of the Royal College of Surgeons, Edinburgh. 8vo, pp. 127. Macphail, Edinburgh, 1845.

We are happy to receive any additions to minute anatomy from so careful and excellent an observer as Mr. J. Goodsir. The work before us contains brief, but in several respects important, notices on many of the intricate structures and processes of the animal body—on nutrition; on the intestinal villi; on absorption and ulceration; on the structure of the human placenta; on the structure and economy of bone, &c.: the principal part of the matter of these papers has already appeared in other publications. Mr. H. Goodsir has also given some contributions derived from zoology, anatomy, and pathology, illustrative of nutrition and secretion.

The powers and properties of nucleated cells have been so often described, that it is only necessary to refer to some of the more important of Mr. Goodsir's remarks. The author fully adopts the views of Dr. M. Barry, who was the first to show the great importance of the nucleus as constituting the active centre from which all cell-formation, and thus indirectly *all* organic formation, proceeds.*

Mr. Goodsir calls these nuclei, together with their cells, "*centres of nutrition*," because they constitute "the permanent source of successive broods of young cells, which from time to time fill the cavity of their parent, and carrying with them the cell-wall of the parent, pass off in certain directions, and under various forms. According to the texture or organ of which the parent forms a part." P. 2.

The author further proposes to employ the term "germinal membrane" in a more extended sense than it has hitherto been used, wishing to signify by it, in fact, the part of all secreting and absorbing surfaces, which, consisting itself of "cells with their cavities flattened," becomes the matrix of those cells which, existing on all free surfaces, constitute the more immediate agents of absorption and secretion. We think the extension here proposed a desirable one, as it tends to place the language of anatomy in harmony with the present views of physiology, according to which the process that gives rise to the embryonic tissues, is correctly regarded as nothing more than a particular instance of the general action of nutrition.

An instance of such a "germinal membrane" is furnished by that thin delicate expansion, called by Bowman the basement membrane, which underlies the epithelium of all mucous surfaces and canals. The following remarks upon this so-called "structureless" membrane are interesting.

"I have always considered the basement membrane, or elementary membrane of glands, as a form of the primary cells of glands, and the source of the secondary or secreting cells, and have therefore been in the habit of naming it primary, or germinal membrane. Mr. Bowman considers it to be simple, or homogeneous. This is true as far as it contains no blood-vessels, and as regards its external or attached layer; but, as in its original condition it consists of cells, and when perfect contains nuclei at equal or variable distances, I do not consider it as simply molecular. These nuclei, or germinal spots, may be certain of the epithelial cells, which become mother cells, between the two layers of the mem-

* Some writers, but of no great authority, have lately questioned the commonly, received opinion, that the nucleus is the first-formed and essential part of the cell, contending that at first each cell is a punctiform vesicle, which grows into a simple cell, and then forms within itself some peculiar substance, either a secretion, or the rudiments of a new cell or nucleus. Whilst numerous facts, and the evidence of so many accurate observers, support the views of M. Barry, we have no inclination to abandon them.—*See the British and Foreign Med. Review. Mr. Paget's Report.*

brane; or cells belonging to the order of the nuclear fibres of Valentin and Henle." P. 3.

The paper on the structure and functions of the intestinal villi is one of much interest, and although the assertion of Mr. Goodsir, that the epithelium is detached at each digestion, has been called in question, we have no doubt of the general truth of his views, and therefore recommend the observations contained in this chapter to the careful notice of our readers.

It is well known that Mr. Cruikshank, in examining with Dr. W. Hunter the intestine of a woman who had died soon after eating a hearty supper, found what they conceived to be the open orifices of the lacteal absorbents. Mr. Goodsir has had an opportunity of inspecting two preparations of the intestine upon which these anatomists made their observations; and he is satisfied, that although the description and figures of lacteals radiating within the villi are quite correct, yet that Cruikshank was led into an error, when he represented those vessels as opening on the free surface of the intestine.

It has been the general opinion of physiologists that absorption and secretion depend on forces of different and opposite tendencies—the one attractive, the other repulsive. Mr. Goodsir contends, on the contrary, that "they are both attractive, absorption being but the first stage in the process of secretion. Secretion, in fact, differs from absorption, not physiologically, but morphologically."

The views of the author upon the agency of the epithelium of the intestine are so well known, that we need here only state that he conceives the cells contained within the villi perform the office of absorbing the chyle, whilst the prismatic epithelium covering the exterior of the villi and the Lieberkühnian follicles becoming detached, cast their contents into the intestine, and so effect secretion. A corroborative fact is mentioned to the effect that when the villi turgid with chyle, have been kept for some time in spirits, the contents of the vesicles become opaque from the coagulation of the albumen they have taken up. It is also stated, which we have ourselves often observed, that on examining an animal just killed, a commencing separation between the nutritious and excrementitious matters is observed in the intestine, a milky substance, consisting, according to the author, of a transparent fluid, with a few oil globules, adhering to the mucous surface, whilst a brownish fluid occupies the cavity of the bowel.

The author's account of the process of ulceration in cartilage, of the separation of dead from living bone, of the detachment of a slough in the soft parts, for these are all effected in the same way, are deserving of attention. In all these actions, the vessels play a subordinate part. The process is thus described:—

"A rapidly-extending ulcerated surface appears as if the textures were scooped out by a sharp instrument. The textures are separated from the external medium by a thin film. This film is cellular in its constitution, and so far it is analogous to the epidermis or epithelium. It is a peculiarly endowed cellular layer, which takes up progressively the place of the subjacent textures, these being prepared for dissolution, either by the state of the system, the condition of the part, or by some influence induced by the contiguity of the new formation." P. 15.

The fifth chapter contains some valuable facts connected with secreting structures; and it is due to Mr. Goodsir to state, that several of the observations now presented in a connected form were published as early as 1842. The principal object is to show, by a series of well-selected observations, that all true secretions, bile, urine, milk, semen, &c., are formed by a vital action of the nucleated cell, and that they are first contained in the cavity of that cell; and further, that growth and secretion are identical—the same vital process under different circumstances. The concurrent testimony of physiologists in all parts of Europe, have firmly established the truth of these two positions, which may now be regarded as fixed principles of the science of organization.

In the paper before us it is asserted that the metamorphoses taking place in the cell, and which constitute the act of secretion, are effected by the nucleus, not by the cell-wall; and, in support of this, an interesting fact is mentioned that the former is, in every instance, directed towards the source of nutritive matter, whilst the latter or cell-wall is opposed to the cavity into which the secretion is cast. It is known that in certain glands the secreting cells are thrown into the excretory passage entire instead of being ruptured: such cells, it is affirmed, after thus ceasing to form a part of the organism, still retain so much individuality of life, as to proceed with their metamorphosing action. The following interesting illustration of these independent powers of cell-life is given by Mr. H. Goodsir: it is found that the component cells of the semen not only undergo a development as they descend in the seminal duct of the decapod crustaceans, but actually experience their ultimate change by which the spermatozoa are produced, after they have been deposited in the spermatheca of the female.

In speaking of the synovial membranes Mr. Goodsir states his belief that their highly vascular fringes, considered by Clopton Havers to be glands for the secretion of the lubricating fluid of the joint, do really perform that office; and we may remark that, an examination of some very successful injections, induce us to agree in the correctness of this opinion.

The results of the author's researches on that difficult branch of minute anatomy, the texture of the lymphatic glands, are thus given.

"1. That the lymphatic glands are merely networks of lymphatic vessels, deprived of all their tunics but the internal, the epithelium of which is highly developed for the performance of particular functions.

"2. That these peculiar lymphatics are supplied with a fine capillary network, to supply matter for the continual renovation of the epithelium." P. 49.

Of these papers we consider the most important to be that on the structure of the human placenta, the whole of which is worthy of a careful perusal. It is difficult, without exceeding the limits of our present notice, to point out the various membranes and other parts connected with the placenta, and especially to describe the principles of their formation and relations, derived from an extended study of ovology, by which alone clear notions of the complex structures, as they exist in the human female, can be obtained.

Mr. Goodsir's observations refer more especially to the true relations and minute texture of the mucous coat of the uterus after conception—to the formation of the decidua—to the texture of the tuft and villi of the placenta—and to the disposition of the fœtal and maternal vessels; in short, to all those points concerning which there has been in late years so much of research and controversy. In considering these intricate questions, we shall take the liberty of reversing the order observed by the author, conceiving that just views are more likely to be conveyed by inquiring first into the several individual parts composing the placenta, and then by investigating that organ in its complete and formed condition.

The outer membrane of the ovum, or the chorion, is, it is well known, the part by which the relations, whatever they may be, existing between the fœtal and maternal systems, are established. This membrane, it must be remembered, is like its analogue in the bird's egg, the membrana testæ, in itself entirely destitute of blood-vessels. It presents a vast multitude of projections, called tufts and villi, each of which, according to Mr. Goodsir, consists of a very delicate and transparent, but at the same time firm membrane, (called by the author the internal membrane of the placental villus). Beneath this membrane are a number of nucleated cells, (the internal cells of the villus,) egg-shaped, difficult to define on account of their transparency, but having nuclei easily recognisable. In the progress of development the ramifications of the umbilical blood-vessels, sustained upon the mucous sack of the allantois, are by that pouch carried up to the chorion, into the tufts and villi of which the ultimate branches penetrate, so as to be received like the fingers placed in a glove.* The umbilical capillary vessels, the minute disposition of which has been so admirably described by Professor Weber and Mr. Dalrymple, are thus brought into contact with the nucleated cells just described, and which, according to Mr. Goodsir, play an important part in the nutrition of the embryo.

We now have to consider the much more obscure parts belonging to the maternal system, and firstly of the decidua. The researches of Professors Weber and Sharpey show that, after impregnation has taken place, the mucous coat of the uterus swells and becomes lax, and more especially that its glands or follicles experience an important change, enlarge, and pour out a semi-fluid, granular secretion. Mr. Goodsir particularly describes, what the above physiologists have not noticed, that part, namely, of the mucous coat of the uterus, which is placed between the follicles: this, in a recently-impregnated specimen, was highly vascular, and the areolæ of the vessels were occupied by a texture consisting entirely of nucleated cells, which by Bär and Wagner were supposed to be uterine papillæ; beneath these cells the uterine surface was covered by a membrane which seemed to be continuous with the basement or germinal membrane of the follicles. The structures now described, the enlarged follicles and interfollicular vascular surface, constitute the *decidua vera*, which is thus a highly-organized glandular and vascular structure.

---

* It would tend to prevent misconception if the terms used by many embryologists were employed; according to which the outer, non-vascular chorion is called *exo-chorion*, and the vascular layer, consisting of the umbilical vessels, the *endo-chorion*.

According to the author, " about the time when the ovum reaches the uterus, the developed mucous membrane or decidua begins to secrete; the os uteri becomes plugged up by this secretion, where it assumes the form of elongated epithelial cells ; the cavity of the uterus is filled with a fluid secretion, the 'hydroperione' of Breschet, and, in the immediate neighbourhood of the ovum, the secretion consists of cells of a spherical form." (P. 58.) It is these cells which, enveloping the ovum, constitute the *decidua reflexa.* " From what has now been stated, it appears, that the decidua consists of two distinct elements : the mucous membrane of the uterus thickened by a peculiar development, and of a non-vascular cellular substance, the product of the uterine follicles." (P. 58.)

The ovum approaches the thickened uterine mucous membrane, or that portion usually described as the decidua serotina; and at this time, when the allantois is conveying the umbilical vessels to the tufts of the chorion, " the vessels of the decidua (which as shown above are the uterine) enlarge, and assume the appearance of sinuses encroaching on the space formerly occupied by the cellular (reflected) decidua, in the midst of which the villi of the chorion are embedded. This increase in the calibre of the decidual capillaries, goes on to such an extent, that finally the villi are completely bound up or covered by the membrane which constitutes the walls of the vessels, this membrane following the contour of all the villi, and even passing to a certain extent over the branches and stems of the tufts. Between this membrane, or wall of the enlarged decidual vessels, and the internal membrane of the villi, there still remains a layer of the cells of the decidua."

Having described the integral parts forming the placenta, it may be stated that, when that organ is examined in the uninjected state, proceeding from its exterior, the following parts are met with :

1. The external membrane of the tufts and villi, a portion of the wall of the vascular system of the uterus, continuous, prior to the detachment, with the rest of that wall.
2. The external cells of the villi, belonging to the decidua.
3. The internal membrane of the villi, consisting of the non-vascular chorion (exo-chorion).
4. The internal cells of the villi, which are cells of the chorion.
5. The umbilical blood-vessels.

It thus appears that the placenta, as Hunter and most anatomists have contended, consists of a fœtal and of a maternal portion intermixed, but essentially distinct from and independent of each other; one part being formed by the membranes and blood-vessels of the fœtus, and the other, of the mucous coat, follicles, and blood-vessels of the uterus. It would be incompatible with our limits to enter further into the details of the relations between the villi of the chorion with the great uterine vessels ; for such details we refer our readers to the work before us, and also to the valuable observations of Professors Weber, Sharpey, and Reid, and of Mr. Dalrymple.

Our present notice would, however, be incomplete if the physiological inferences of Mr. Goodsir were not briefly mentioned. The great object of this gentleman is to show, what we have ourselves long held with reference to the umbilical vesicle, that the placenta accomplishes the nutrition of the fœtus, independently of its respiratory office, upon exactly the same principles as those effecting the ordinary process of chylous absorption. The author concludes that the external cells of the placental villi separate from the blood of the mother, the matter destined for the blood of the fœtus, being thus at once absorbing and secreting cells and performing, during intra-uterine existence, a function for which is substituted in extra-uterine life the digestive action of the gastro-intestinal mucous membrane: that the internal cells of the placental villi absorb, through the non-vascular chorion, the matter secreted as above, performing a function for which is substituted in extra-uterine life the action of the absorbing chyle cells of the intestinal villi. This account shows, what is proved in various other ways, that there is no passage of blood from the mother to the child; it is merely nutritive matter which is deposited from the uterine vessels and which, after the elaboration just described, is taken up by the capillaries of the umbilical blood-vessels.

There is one error into which the author has fallen which it is desirable to correct, as it would otherwise introduce confusion where in reality none ought to exist. Mr. Goodsir says, "I am inclined to look upon the cellular (reflected) decidua, as representing in the gestation of the mammal the albumen of the egg of the oviparous animal. They are both supplied by a certain portion of the oviduct, and they are both brought into play after the nourishment supplied by the ovary is exhausted, or in the course of being exhausted." (P. 59). All the evidences of comparative ovology are opposed to this opinion. In the first place, the ovum of the mammal is provided, like that of the bird, with albumen placed without the yelk: in the second place, the albumen of the fowl is secreted at the upper division of the oviduct, above the isthmus that is to say, whilst the decidua is formed in the uterus, to which the part of the oviduct secreting the shell corresponds in the bird; and, lastly, there is abundant proof to show that, in all the earlier stages of development, and especially as regards the nourishment of the embryo by the contents of the umbilical vesicle (into which in all animals the white passes to mix with the yelk) and its vessels, the omphalo-mesenteric, there is a perfect identity between the mammal and the bird.

The papers contributed by Mr. Harry D. S. Goodsir are of interest, especially that "On the Anatomy and Development of the Cystic Entozoa." From the account given of the reproductive process in the *Acephalocyst*, it appears to arise, like all other organic beings, from a cell or vesicle, which is attached to the middle of the three membranes composing the parent hydatid, and which is therefore called "the germinal membrane." Shortly before the young hydatid separates from the parent, smaller cells are seen within it, and in this manner the successive and multitudinous progeny is generated, and these escaping by the rupture of the original cell, each attain a proper habitat, and then, but not till then, begins to throw off its young from the germinal membrane.

We regret that our remarks have already extended so far, that we cannot notice the other papers contained in this valuable work, which must be regarded as a most acceptable addition to all who are engaged in minute anatomy and physiology.

